# Circulating Th22 and Th9 Levels in Patients with Acute Coronary Syndrome

**DOI:** 10.1155/2013/635672

**Published:** 2013-12-23

**Authors:** Ying-zhong Lin, Bang-wei Wu, Zheng-de Lu, Ying Huang, Ying Shi, Hao Liu, Ling Liu, Qiu-tang Zeng, Xiang Wang, Qing-wei Ji

**Affiliations:** ^1^Department of Cardiology, The People's Hospital of Guangxi Zhuang Autonomous Region, Nanning, China; ^2^Institute of Cardiovascular Diseases, Union Hospital, Tongji Medical College, Huazhong University of Science and Technology, Wuhan, China; ^3^Department of Ultrasound, The People's Hospital of Guangxi Zhuang Autonomous Region, Nanning, China

## Abstract

*Background*. CD4+ T helper (Th) cells play critical roles in the development and progression of atherosclerosis and the onset of acute coronary syndromes (ACS, including acute myocardial infarction (AMI) and unstable angina pectoris (UAP)). In addition to Th1, Th2, and Th17 cells, Th22 and Th9 subsets have been identified in humans. In the present study, we investigated whether Th22 cells and Th9 cells are involved in the onset of ACS. *Methods*. The frequencies of Th22 and Th9 cells were detected using a flow cytometric analysis and their related cytokine and transcription factor were measured in the AMI, UAP, stable angina pectoris (SAP), and control groups. *Results*. The results revealed a significant increase in the peripheral Th22 number, AHR expression, and IL-22 levels in patients with ACS compared with those in the SAP and control groups. Although there was no difference in the peripheral Th9 number among the four groups, the PU.1 expression and IL-9 levels were significantly increased in patients with ACS compared with the SAP and control groups. *Conclusions*. Circulating Th22 and Th9 type responses may play a potential role in the onset of ACS symptom.

## 1. Introduction 

CD4+ T helper (Th) cells play major roles in the inflammatory process of atherosclerosis and the onset of acute coronary syndromes (ACS) including unstable angina pectoris (UAP) and acute myocardial infarction (AMI) [1–3]. CD4+ Th cells, accompanied by macrophages and dendritic cells, are easily detected in the shoulders of plaque and lead to thrombosis, embolization, and varying degrees of obstruction of myocardial perfusion [4–6]. CD4+ Th cells include effector T cells, which protect against pathogens, and regulatory T cells (Tregs), which protect against effector responses to autoantigens and against responses to exogenous antigens that are harmful to the host. Based on their cytokine secretion profile, effector T cells are functionally subdivided into three types, Th1, Th2, and Th17. The roles of four CD4+ Th cell lineages, Th1, Th2, Th17, and Treg cells, in atherosclerosis are widely studied [1, 2]. During the past two decades, the Th1/Th2 paradigm prevailed, and Th1 cells were thought to promote the pathology in atherosclerosis and Th2 cells were thought to attenuate the disease [7–9]. Recently, Th17, the third subpopulation of Th cells, was found to promote atherosclerotic lesion development in atherosclerotic-prone models and it was related to the onset of coronary artery disease in a number of studies [10–12]. The role of Th17 in atherosclerosis remains uncertain. Although identical models are used by different experimental groups, the results are inconclusive in the following studies: Danzaki et al. found that IL-17 deficiency accelerated unstable plaque formation; Butcher et al. found that IL-17 deficiency reduced atherosclerotic plaques; and Madhur et al. found that there was no correlation between IL-17 deficiency and atherosclerotic plaques in ApoE−/− mice [13–16]. The hypothesis that Tregs play a protective role against atherosclerosis development is well accepted [17–19]. Tregs effectively maintain self-tolerance and dampen inflammatory processes by inhibiting Th1, Th2, and Th17 responses through cell-cell contact and secretion of cytokines, including IL-10, IL-35, and TGF-*β*. There is a hypothesis that neither the imbalance of Th1/Th2 nor the imbalance of Th17/Treg, but the imbalance between the effector T cells and Tregs, plays a critical role in atherosclerosis [20, 21].

Th9 and Th22, two additional subsets of effector T cells, have recently been described [22–26]. Th9 cells predominantly produce IL-9, which was previously known as a Th2-derived cytokine, and Th22 cells primarily produce IL-22, which was previously known as a Th17-derived cytokine. There is accumulating evidence that Th9 and Th22 cells are highly involved in human inflammatory and autoimmune diseases [27–30]. More recently, evidence demonstrated an upregulated Th22 response and increased IL-9 levels involved in atherosclerosis and associated with the onset of carotid plaque symptom [31–33]. However, whether Th22 and Th9 responses are related to the onset of ACS has not been investigated. In this study, we measured the frequencies of Th22 and Th9, their transcription factors, aryl hydrocarbon receptor (AHR) and PU.1, and the IL-22 and IL-9 levels in the stable angina pectoris (SAP), UAP, AMI, and control groups.

## 2. Methods

### 2.1. Patients

A total number of 83 patients were enrolled in the present study, which includes four groups: (1) the SAP group (13 men and 5 women, mean age 61.6 ± 12.3), inclusion criteria: typical exertional chest discomfort that was associated with downsloping or horizontal ST-segment depression >1 mm in an exercise test; (2) the UAP group (17 men and 6 women, mean age 63.8 ± 10.2), inclusion criteria: chest pain at rest with definite ischemic electrocardiographic changes: ST-segment changes and/or T-wave inversions; (3) the AMI group (15 men and 5 women, mean age 64.3 ± 11.0), inclusion criteria: myocardial infarction that was confirmed by a significant increase of troponin I and creatine kinase MB levels; (4) the control group, which consisted of 22 subjects with normal coronary artery (15 men and 7 women, mean age 61.4 ± 8.9).

Informed consents were obtained from all cases and the study protocol conforms to the ethical guidelines of the Declaration of Helsinki Principles (revised in Edinburgh 2000) as reflected in a priori approval by the institution's human research committee. Patients with valvular heart disease, thromboembolism, collagen disease, disseminated intravascular coagulation, advanced liver disease, renal failure, malignant disease, or septicemia or those who were on steroid therapy were excluded from the study.

### 2.2. Blood Samples

In the AMI group, blood samples were immediately obtained upon admission to the hospital. Blood samples were obtained from the other patients on the morning following admission; the patients were in a fasting state in a recumbent position, and the blood was drawn with a 21-gauge needle for clean venipuncture of an antecubital vein. The samples were collected into sodium heparin vacutainers (Becton-Dickinson). The peripheral blood mononuclear cells (PBMCs) were prepared by Ficoll density gradient for analysis by flow cytometric analysis and real-time quantitative reverse transcription-polymerase chain reaction (RT-PCR). The plasma obtained after centrifugation was stored at −80°C until further use.

### 2.3. Flow Cytometric Analysis of Th9 and Th22 

#### 2.3.1. Cell Preparation

The PBMCs were suspended at a density of 2 × 10^6^ cells/mL in a complete culture medium (RPMI 1640 supplemented with 100U/mL penicillin, 100 *μ*g/mL streptomycin, 2 mM glutamine, and 10% heat-inactivated fetal calf serum, Gibco BRL). The cell suspension was transferred to each well of 24-well plates. The cultures were stimulated with phorbol myristate acetate (PMA, 25 ng/mL) plus ionomycin (1 *μ*g/mL) (Alexis Biochemicals, San Diego, CA) for 4 h, in the presence of 1 *μ*L monensin (Alexis Biochemicals, San Diego, CA). The incubator was set at 37°C under a 5% CO_2_ environment. After 4 h of culture, the contents of the well were transferred to 5 mL sterile tubes. The cells were centrifuged at 1500 rpm for 5 min.

#### 2.3.2. Surface and Intracellular Staining

The cells were aliquoted into tubes and washed once in phosphate-buffered saline (PBS). The cells were incubated with FITC anti-human CD4 (eBioscience, San Diego, CA) in room temperature for 20 min. After the surface staining, the cells were stained with Alexa anti-human IL-9 and phycoerythrin (PE) anti-human IL-22 for Th9 and Th22 detection (Both antibodies are from eBioscience, San Diego, CA). In the present study, CD4+ IL9+ IL-22− was considered to be the Th9 cell, while CD4+ IL22+ IL-9− was considered to be the Th22 cell. Isotype controls enabled correct compensation and confirmed the antibody specificity. The stained cells were analyzed by flow cytometric analysis using a FACScan cytometer equipped with CellQuest software (BD Bioscience Pharmingen).

### 2.4. Real-Time Quantitative Reverse Transcription-Polymerase Chain Reaction (RT-PCR)

The total RNA was extracted with TRIzol (Invitrogen, USA), according to the manufacturer's instructions. The cDNA was synthesized with random hexamer primers and RNase H-reverse transcriptase (Invitrogen). TaqMan primers and probes for human AHR and PU.1 were purchased from Biosune Biotechnology. The following primer pairs were used: AHR: F: 5′-CTTCCAAGCGGCATAGAGAC-3′, R: 5′-AGTTATCCTGGCCTCCGTTT-3′ (198 bp), PU.1: F: 5′-CCAGCTCAGATGAGGAGGAG-3′, R: 5′-CAGGTCCAACAGGAACTGGT-3′ (152 bp). The quality of cDNA subjected to the RT-PCR was controlled by amplification of transcripts of GAPDH. GAPDH was analyzed using the following primers: F: 5′-GAGTCAACGGATTTGGTCGT-3′, R: 5′-GACAAGCTTCCCGTTCTCAG-3′ (185 bp). Quantitative PCR was performed on ABI PRISM 7900 Sequence Detector system (Applied Biosystems) using SYBR Green I Assay (Takara Biotechnology). Relative gene expression level (the amount of target, normalized to endogenous control gene) was calculated using the comparative Ct method formula 2^−ΔΔCt^.

### 2.5. ELISA Detection of the Levels of IL-9 and IL-22

The levels of IL-9 and IL-22 were measured by enzyme-linked immunosorbent assay (ELISA), following the manufacturer's instructions (eBioscience, San Diego, CA). The minimal detectable concentrations were 0.5 pg/mL for IL-9 and 5 pg/mL for IL-22. The intra-assay and interassay coefficients of variation for all ELISA were <5% and <10%, respectively. All the samples were measured in duplicate.

### 2.6. Gensini Score

The severity of coronary stenosis in the patients was estimated with a Gensini coronary score following coronary angiography. The Gensini score was computed by assigning a severity score to each coronary stenosis according to the degree of luminal narrowing and its geographic importance. The reduction in the lumen diameter and the roentgenographic appearance of concentric lesions and eccentric plaques were evaluated (reductions of 25%, 50%, 75%, 90%, and 99% and complete occlusion were assigned Gensini scores of 1, 2, 4, 8, 16, and 32, resp.). The score was multiplied by a factor that incorporates the importance of the position of the lesion in the coronary arterial tree as follows: 5 for the left main coronary artery; 2.5 for the proximal left anterior descending coronary artery (LAD) or left circumflex artery (LCX); 1.5 for the mid-LAD; and 1 for the distal LAD, the right coronary artery, or the mid-distal LCX.

### 2.7. Statistical Analysis

All of the data were given as the mean ± SD. The data were analyzed by ANOVA. When significance was found, a Newman-Keuls test was performed as a post-hoc analysis to detect the difference among groups. Spearman's correlation was used to calculate correlations between two continuous variables. In all tests a value of *P* < 0.05 was considered to be statistically significant.

## 3. Results

### 3.1. Baseline Characteristics

There was no significant difference in age, gender, or history of hypertension, diabetes, or tobacco use in these four groups. The levels of low-density lipoprotein cholesterol (LDL-C), fasting glucose, C-reactive protein (CRP), left ventricular end-diastolic dimension (LVEDD), and the Gensini score were significantly higher in the AMI group than the control group, whereas the left ventricular ejection fraction (LVEF), in the AMI and UAP groups was lower than that of the control group. The other parameters including prehospital medications are listed in Table 1.

### 3.2. Circulating Th22 and Th9 Frequencies

**Figure 1:**
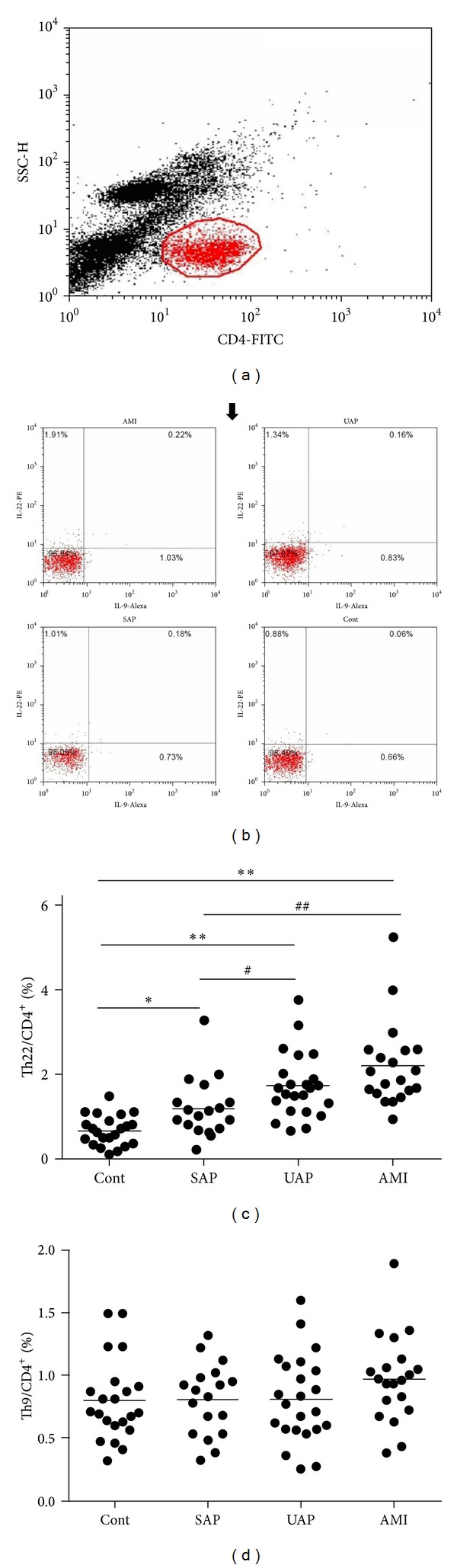
Circulating Th22 and Th9 frequencies in each group. (a) CD4+ T cells were gated by flow cytometry. (b) Representation of intracellular cytokine staining of Th22 and Th9 from each group. (c) The frequencies of Th22 were markedly higher in patients with acute myocardial infarction (AMI), unstable angina pectoris (UAP), and stable angina pectoris (SAP) than those in the control group (Cont). (d) The frequencies of Th9 showed no differences among these groups. *P < 0.05 versus control, **P < 0.01 versus control, ^#^ *P* < 0.05 versus SAP group, and ^##^ *P* < 0.01 versus SAP group.

As shown in Figure 1, the frequencies of Th22 (CD4+ IL-22+ IL-9−/CD4+ T cells) were markedly higher in patients with AMI (2.19 ± 0.99%), UAP (1.72 ± 0.76%), and SAP (1.17 ± 0.67%) than those in the control group (0.67 ± 0.36%). The frequencies of Th9 (CD4+ IL-9+ IL-22−/CD4+ T cells) showed no obvious difference among the AMI group (0.97 ± 0.34%, *P* = 0.08), UAP group (0.82 ± 0.34%), SAP group (0.78 ± 0.28%), and control group (0.71 ± 0.27%). Furthermore, 83 cases were divided into a hypertensive group (48 cases) and a normotensive group (35 cases), or a diabetic group (19 cases) and a nondiabetic group (64 cases). The results showed that there was no significant difference in the frequencies of Th22 and Th9 between the hypertensive group (1.77 ± 1.02%, 0.88 ± 0.31%, resp.) and the normotensive group (1.64  ±  0.70%, 0.85  ±  0.34%, resp.) and between the diabetic group (1.70 ± 0.94%, 0.87 ± 0.34%, resp.) and the nondiabetic group (1.77 ± 0.80%, 0.84 ± 0.29%, resp.).

### 3.3. Expression of AHR and PU.1

**Figure 2:**
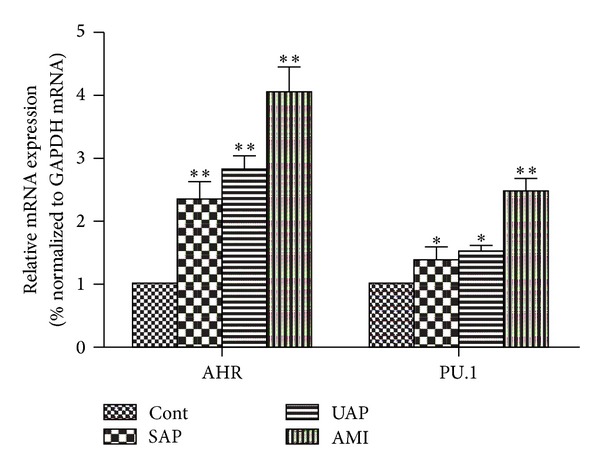
Expression of AHR and PU.1. A: the expression of AHR was markedly higher in the acute myocardial infarction (AMI), unstable angina pectoris (UAP), and stable angina pectoris (SAP) groups than in the control group (Cont). B: the expression of PU.1 was markedly higher in the AMI, UAP, and SAP groups than in the control group. *P < 0.05 versus Control; **P < 0.01 versus control.

As shown in Figure 2, the expression of AHR and PU.1 was markedly higher in the AMI (4.06 ± 0.96, 2.47 ± 0.48, resp.), UAP (2.82 ± 0.55, 1.51 ± 0.27, resp.) and SAP (2.35 ± 0.67, 1.38 ± 0.51, resp.), groups than in the control group.

### 3.4. Cytokines Concentrations Analysis

**Figure 3:**
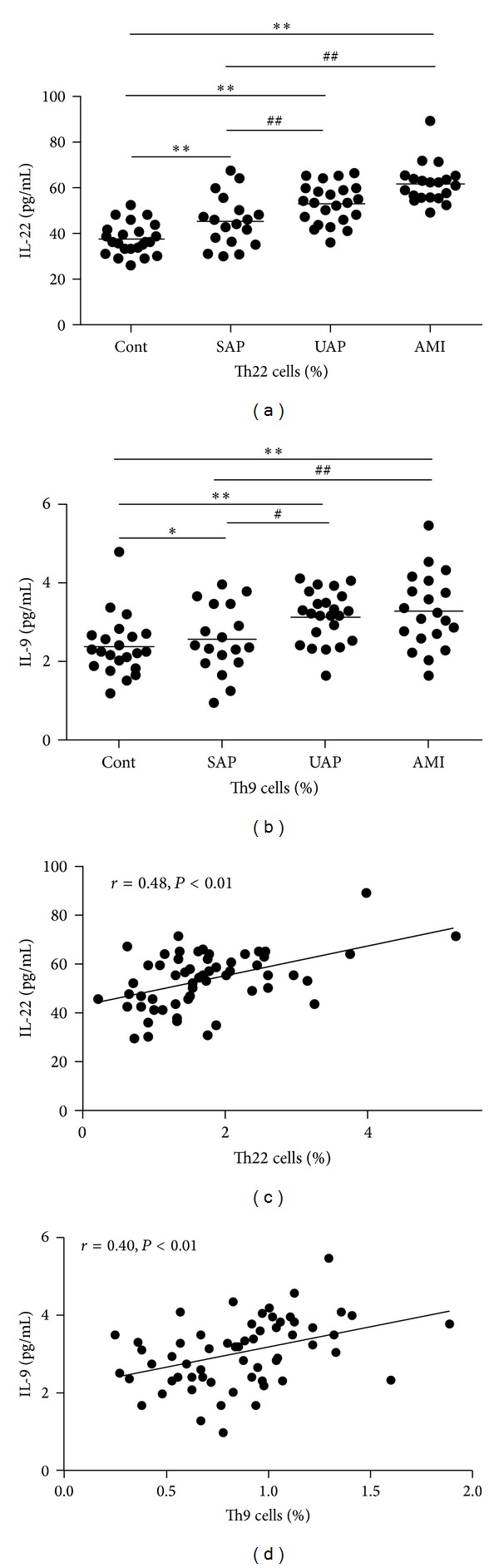
Plasma IL-22 and IL-9 concentrations analysis. (a) The plasma IL-22 levels in patients with acute myocardial infarction (AMI) and unstable angina pectoris (UAP) were significantly increased compared with those of the control group (Cont) and the stable angina pectoris (SAP) group; the plasma IL-22 levels in patients with SAP were significantly increased compared with those of the control group. (b) The plasma IL-9 levels in patients with AMI and UAP were significantly increased compared with those of the control group and the SAP group; the plasma IL-9 levels in patients with SAP were significantly increased compared with those of the control group. (c) The IL-22 concentrations showed a positive correlation with the frequencies of Th22 cells. (d) The IL-9 concentrations showed a positive correlation with the frequencies of Th9 cells. *P < 0.05 versus control, **P < 0.01 versus control, ^#^ *P* < 0.05 versus SAP group, and ^##^ *P* < 0.01 versus SAP group.

As shown in Figure 3, the plasma IL-22 and IL-9 levels in patients with AMI (61.67 ± 8.77 pg/mL, 3.29 ± 0.94 pg/mL, resp.) and UAP (52.93 ± 8.64 pg/mL, 3.15 ± 0.65 pg/mL, resp.) were significantly increased compared with those of the control group (37.41 ± 7.01 pg/mL, 2.40 ± 0.75 pg/mL, resp.) and the SAP group (45.06 ± 11.12 pg/mL, 2.57 ± 0.86 pg/mL, resp.), while the plasma IL-22 and IL-9 levels in patients with SAP were significantly increased compared with those of the control group. The IL-22 concentrations showed a positive correlation with the frequencies of Th22 cells (*r* = 0.48, *P* < 0.01, Figure 3(c)) and the IL-9 concentrations showed a positive correlation with the frequencies of Th9 cells (*r* = 0.40, *P* < 0.01, Figure 3(d)). There was no correlation between the IL-22 concentrations and the frequencies of Th9 cells (*r* = 0.11, *P* > 0.05) and between the IL-9 concentrations and the frequencies of Th22 cells (*r* = 0.02, *P* > 0.05).

### 3.5. Spearman's Correlation Analysis

We assessed whether the frequencies of Th22 and Th9 were associated with lipid and lipoprotein fractions, fasting glucose, creatinine, CRP, and the Gensini score which was used to quantify the severity of coronary artery stenosis in patients with coronary artery disease (CAD). As shown in Table 2, the frequencies of Th22 were positively correlated with TC, TG, LDL-C, Apo B, fasting glucose, and CRP in patients with CAD. The results also showed that the frequencies of Th9 were positively correlated with TC, LDL-C, and CRP in patients with CAD. There was no correlation between  the frequencies of Th22 and Th9 and the Gensini score. Because LVEF and LVEDD are associated with the short- and long-term prognosis in ACS, the correlation betweenthe frequencies of Th22 and Th9 and the levels of LVEF and LVEDD in ACS was analyzed. We found that both Th22 and Th9 numbers were positively correlated with LVEDD but negatively correlated with the LVEF in patients with ACS.

## 4. Discussion

CD4+ T cells differentiate into a variety of effector subsets including Th1, Th2, Th17, and the more recently identified Th9 and Th22 cells, which selectively express IFN-*γ*, IL-4, IL-17, IL-9, and IL-22, respectively. In previous studies, we and others have found that changes in Th1, Th2, and Th17 are associated with the onset of ACS [3, 7, 10, 21]. In this study, we first found that the peripheral blood Th22 number, AHR expression, and plasma IL-22 levels were significantly higher in patients with ACS compared with the SAP and control groups. There were no differences in the frequencies of Th9 in the ACS patients and in the control group patients, but its transcription factor PU.1 expression and functional cytokine IL-9 levels were significantly increased in patients with ACS. A correlation analysis showed that the peripheral blood Th22 number was positively correlated with the levels of IL-22, and the peripheral blood Th9 number was positively correlated with the levels of IL-9. The results of this study suggest that the frequencies of Th22 and Th9 are moderately positive with the lipid and lipoprotein fractions, fasting glucose, and CRP and are related to the myocardial contractile function and remodeling. Notably, the frequencies of Th22 and Th9 showed no correlation with the severity of coronary stenosis measured by the Gensini score in the CAD patients, suggesting that the change is associated with the inflammatory status and plaque destabilization of CAD but not with the severity of the narrowness of the coronary artery.

Th22, the novel subpopulation of the effector CD4+ T cells, was identified in 2009 and is characterized by the production of IL-22, but not of IL-17 or IFN-*γ* [24, 25]. IL-22 was also secreted by activated Th1, Th17, NK cells, NKT cells, and lymphoid tissue inducer cells. It is well known that Th1 differentiation is induced by IL-12 and requires the lineage-specifying transcription factor T-bet and Th2 differentiation is induced by IL-4 and requires transcription factor GATA-3, whereas Th17 differentiation is induced by IL-6 plus TGF-*β* and requires the transcription factors RORc in human or ROR*γ*t in mice. Duhen and Trifari found that Th22 differentiation is induced by IL-6 alone or plus TNF-*α* and requires the transcription factor AHR and is modulated by RORc in humans [24, 25]. Th1 cells express CXCR3 and CCR5, Th2 cells express CCR3 and CCR4, Th17 cells express CCR6, and these cells do not express CCR10, which is highly expressed by Th22 cells. More recently, Th22 was identified in mice. Basu et al. found that although IL-22 was secreted by various types of cells, Th22 (CD4+ IL-22+ T) cells were the critical source of IL-22 during the later stages of inflammation, indicating that Th22 may be essential for controlling chronic inflammation [26]. The effector cytokine of Th22 cells is IL-22, which belongs to the IL-10 cytokine family and binds to the heterodimeric receptor complex consisting of the IL-10 receptor (IL-10R) *β* chain and IL-22R. Because IL-22R is expressed almost exclusively on nonimmune cells, IL-22 acts primarily on tissue cells such as epithelial cells and smooth muscle cells [34]. After binding to its receptor complex, IL-22 activates numerous signaling pathways including the Janus kinase/signal transducers and activators of transcription (JAK/STAT) pathway, predominantly STAT3, and the three major MAPK pathways [35]. IL-22 is upregulated in a number of chronic inflammatory and autoimmune diseases, and the exact role of IL-22 appears to depend on specific inflammatory microenvironments: the protective role of IL-22 has been found in a myocarditis model, whereas the inflammatory role of IL-22 has been demonstrated in rheumatoid arthritis [34, 36].

The role of the Th22 type response in human inflammatory and autoimmune diseases has been reported in a number of studies in recent years. Elevated Th22 type responses were found in human rheumatoid arthritis, systemic sclerosis, systemic lupus erythematosus, and cancer and may be related to the poorer survival rates observed in gastric cancer [29, 30, 37, 38]. There were some studies that initially revealed the role of Th22/IL-22 in atherosclerosis [31, 32]. In immunocytochemical staining with an antibody against IL-22, an abundance of IL-22 was detected in a human carotid plaque [31]. It is notable that IL-22 expression is 7.15-fold higher in symptomatic compared with asymptomatic patients and is positively correlated with CXCL-9, -10, and -11 that are associated with the Th1-like diseases [31, 39]. Recently, T lymphocytes were isolated from carotid plaques and then expanded for measurement by flow cytometry [32]. The results showed that the frequencies of Th22 (CD4+ IL-22+) were approximately 2.2% and the frequencies of IFN-*γ*+ IL-22+ T cells and IL-17+ IL-22+ T cells were approximately 2.9% and 1.8%, respectively. In accordance, the mRNA expression of AHR was elevated in carotid plaques. Taken together, the results indicate that Th22 response is pivotal in the development of atherosclerosis and the onset of acute coronary syndrome by virtue of the secretion of IL-22. It is well known that the JAK/STAT pathway and MAPK pathways participate in the growth and migration of smooth muscle cells, the induction of oxidative stress, the expression of numerous proinflammatory genes, cell injury, and cell death that play a critical role in atherosclerosis [40–42]. IL-22 may be involved in atherosclerosis by the JAK/STAT pathway and MAPK pathways. An investigation is required to understand the properties of Th22 in atherosclerosis.

It is known that Th2 cells are generated in the presence of IL-4, and regulatory T (Treg) cells are generated in the presence of TGF-*β*. IL-4 plus TGF-*β* induce the generation of a novel subset of T cells that produce an abundance of IL-9, designated as Th9 cells [22, 23]. Th9 cells do not have regulatory properties, but they promote inflammation, although they secrete considerable IL-10, which has been considered to be an important mediator in controlling inflammation and immune responses. PU.1 has been recently identified as the lineage-specifying transcription factor of Th9 in humans and mice [43]. PU.1 expression effectively inhibits the production of Th2 related cytokines such as IL-4 and IL-5 and promotes a Th2-to-Th9 switch in vitro. IL-9 has been described as the effector cytokine of Th9 and is associated with immune regulation. In addition to Th9, many types of cells such as Th2, Th17, Treg, and mast cells can secrete IL-9 [44, 45]. Unlike IL-22R, IL-9R is expressed on immune cells such as macrophages, lymphocytes, and dendritic cells. Consistent with the expression of its receptor on multiple cell types, IL-9 has diverse biological effects. A number of studies showed that IL-9 has anti-inflammatory properties, whereas other studies found that IL-9 effectively promoted inflammation. The JAK/STAT pathway plays an essential role in the signal transduction of IL-9. Activation of STAT factors, such as STAT1 and STAT3, was shown to be responsible for the IL-9-induced proliferation of T cells [45].

The Th9 response has been shown to be involved in the pathogenesis of a wide variety of inflammatory diseases such as asthma and experimental autoimmune encephalomyelitis [22, 27, 44, 46]. Staudt et al. found that transferred Th9 cells in mice promoted airway resistance, increased the numbers of eosinophils, and exacerbated asthma that has been considered as a Th2-mediated disease; deficiency in Th9 cells can protect mice from asthma, suggesting that the Th9 response is detrimental in asthma cases [46]. Th9 cells were shown to promote central nervous system inflammation and intestinal inflammation, although whether these results are because of the direct effect of Th9 is unknown [22, 27]. The antitumor effects of the Th9 response have been elucidated in recent studies [47]. More recently, Gregersen et al. found that both circulating and local plaque IL-9 levels were significantly increased in patients with coronary and carotid atherosclerosis, and IL-9 and IL-9R coexpressed in atheroma T cells, suggesting the IL-9/IL-9R axis is involved in the atherosclerotic process [33]. Although there was no significant difference in the peripheral blood Th9 number in each group, the results reveal a rising trend in Th9 levels in patients with ACS (AMI versus control, *P* = 0.08), accompanied by the increase of the PU.1 expression and the plasma levels of IL-9. Taken together, these findings strongly suggest that Th9 responses take part in the development of atherosclerosis and the occurrence of ACS.

## 5. Conclusions

The results of our study first demonstrated that Th22 and Th9 responses were upregulated in patients with ACS, suggesting that change may be associated with plaque destabilization and the onset of ACS. There are some limitations in the present study. Beyond Th22, other subsets of CD4+ T cells such as Th1 and Th17 also secret IL-22, and IL-9 is secreted by many types of CD4+ T cells such as Th2, Th17, and Treg in addition to Th9. Therefore, negative staining for IFN-*γ*, IL-4, IL-17, and FOXP3 may favor to obtain more pure Th22 and Th9 cells. In addition, because of the small sample size, we need to prove our conclusion in a larger scale of the population and research investigations that contribute to the clarification of the role of Th22 and Th9 responses in the atherosclerotic process and the onset of ACS.

## Figures and Tables

**Table 1:** Clinical characteristics of patients.

Characteristics	Control (*n* = 22)	SAP (*n* = 18)	UAP (*n* = 23)	AMI (*n* = 20)
Age (years)	61.4 ± 8.9	61.6 ± 12.3	63.8 ± 10.2	64.3 ± 11.0
Sex (male/female)	15/7	13/5	17/6	15/5
Hypertension, *n* (%)	11 (50.0)	12 (66.6)	15 (65.2)	10 (50.0)
Diabetes, *n* (%)	4 (18.1)	4 (22.2)	5 (21.7)	6 (30.0)
Tobacco, *n* (%)	3 (13.6)	6 (33.3)	4 (17.3)	5 (25.0)
TC (mmol/L)	4.41 ± 0.82	4.09 ± 1.27	4.54 ± 1.41	4.66 ± 1.31
TG (mmol/L)	1.39 ± 0.58	1.53 ± 0.71	1.80 ± 0.76	1.58 ± 0.75
LDL-C (mmol/L)	2.83 ± 0.79	2.74 ± 1.10	2.92 ± 1.20	3.13 ± 1.12*
HDL-C (mmol/L)	1.27 ± 0.18	1.02 ± 0.27	1.08 ± 0.36	1.07 ± 0.22
Apo A (mmol/L)	1.36 ± 0.13	1.18 ± 0.18	1.22 ± 0.32	1.15 ± 0.23
Apo B (mmol/L)	0.95 ± 0.29	0.88 ± 0.24	0.97 ± 0.36	0.99 ± 0.27
GLU (mmol/L)	5.12 ± 0.50	5.51 ± 1.19	5.71 ± 1.54	6.44 ± 2.53*
Creatinine (*µ*mol/L)	81.00 ± 13.21	98.33 ± 29.30	91.13 ± 16.91	76.25 ± 20.36
CRP (mg/L)	1.27 ± 0.35	2.44 ± 1.67	3.57 ± 2.07*	3.87 ± 2.27*
LVEF (%)	63.31 ± 5.26	62.55 ± 9.09	59.30 ± 8.37*	49.40 ± 10.89*
LVEDD (mm)	48.00 ± 1.77	48.05 ± 2.38	48.48 ± 2.85	51.80 ± 3.41*
Gensini score	0	29.67 ± 9.96*	62.23 ± 29.18*	71.15 ± 32.26*
Medications, *n* (%)				
*β*-Blockers	3 (13.6)	4 (22.2)	7 (30.4)	4 (20)
ACEI/ARB	2 (9.1)	3 (16.7)	5 (21.7)	4 (20)
CCB	7 (31.8)	8 (44.4)	10 (43.5)	6 (30)
Nitrates	3 (13.6)	6 (33.3)	9 (39.1)	2 (10)
Statins	2 (9.1)	6 (33.3)	7 (30.4)	5 (25)
Aspirin	6 (27.3)	9 (50)	14 (60.9)	10 (50)

The data are given as the mean ± SD or number of patients. SAP: stable angina; UAP: unstable angina; AMI: acute myocardial infarction; TC: total cholesterol; TG: total triglycerides; LDL-C: low-density lipoprotein cholesterol; HDL-C: high-density lipoprotein cholesterol; GLU: fasting glucose; CRP: C-reactive protein; LVEF: left ventricular ejection fraction; LVEDD: left ventricular end-diastolic dimension; ACEI: angiotensin-converting enzyme inhibitor; ARB: angiotensin receptor blocker; CCB: calcium channel blocker.

*P < 0.05 versus control.

**Table 2:** Spearman's correlation of the frequencies of Th22 and Th9 with cardiovascular risk factors.

	Th22/CD4+ T cells (%)	Th9/CD4+ T cells (%)
TC (mmol/L)	0.45**	0.26*
TG (mmol/L)	0.39**	0.08
LDL-C (mmol/L)	0.27*	0.27*
HDL-C (mmol/L)	0.06	0.07
Apo A (mmol/L)	−0.04	−0.02
Apo B (mmol/L)	0.36*	0.06
GLU (mmol/L)	0.30*	0.01
Creatinine (*μ*mol/L)	−0.14	−0.03
CRP (mg/L)	0.35**	0.33**
Gensini score	0.23	0.06
LVEF (%)	−0.36**	−0.35*
LVEDD (mm)	0.41**	0.37**

*P < 0.05; **P < 0.01.

## Abbreviations

ACS: Acute coronary syndrome
ACEI: Angiotensin-converting enzyme inhibitor
AMI: Acute myocardial infarction
ARB: Angiotensin receptor blocker
CCB: Calcium channel blocker
CAD: Coronary artery disease
CRP: C-reactive protein
GLU: Fasting glucose
HDL-C: High-density lipoprotein cholesterol
LDL-C: Low-density lipoprotein cholesterol
LVEDD: Left ventricular end-diastolic dimension
LVEF: Left ventricular ejection fraction
PBMCs: Peripheral blood mononuclear cells
RT-PCR: Real-time quantitative reverse transcription-polymerase chain reaction
SAP: Stable angina
TC: Total cholesterol
TG: Total triglycerides
UAP: Unstable angina.
